# A Preclinical Study of a PSMA Ligand-Based Dual-Modality Probe for Radical Prostatectomy

**DOI:** 10.3390/ph19040564

**Published:** 2026-04-01

**Authors:** Haoxi Zhou, Zhiqiang Chen, Long Yi, Baojun Wang, Shaoxi Niu, Yu Gao, Xu Zhang

**Affiliations:** 1 Department of Urology, The Third Medical Center, Chinese PLA General Hospital, Beijing 100089, China; drhx_chow@163.com (H.Z.);; 2 Chinese PLA Medical School, Chinese PLA General Hospital, Beijing 100853, China

**Keywords:** prostate-specific membrane antigen, [^68^Ga]Ga-PSMA-DF, dual-modality imaging, PET/CT, fluorescence imaging, radical prostatectomy

## Abstract

**Purpose**: Prostate-specific membrane antigen (PSMA) is a well-established molecular target in prostate cancer (PCa). Both radionuclide imaging and near-infrared fluorescence (NIRF) imaging offer high sensitivity for in vivo tumor detection. PSMA-targeted dual-modality probes integrating these two imaging techniques provide complementary preoperative and intraoperative tumor visualization, thereby improving surgical guidance in PCa. In this study, we aimed to develop a novel dual-labeled PSMA probe combining radioactive and fluorescent properties to achieve precise tumor delineation during radical prostatectomy (RP). **Methods**: A high-affinity PSMA-targeted fluorescent probe (PSMA-DF) was synthesized using solid-phase synthesis. Subsequent radiolabeling with the radionuclide [^68^Ga]Ga yielded the successful generation of a dual-modal PSMA-targeted molecular probe, namely [^68^Ga]Ga-PSMA-DF. The probe was systematically evaluated both in vitro and in vivo, and its safety profile was assessed through acute toxicity testing. Tumor-bearing nude mouse models were established using PSMA-positive 22Rv1 and PSMA-negative PC-3 PCa cell lines. Imaging performance, tumor-targeting specificity, and biodistribution of the probe were comprehensively evaluated using micro-PET imaging, in vivo fluorescence imaging, and biodistribution studies. **Results**: High-quality and high-purity PSMA-DF was successfully prepared, which exhibited excellent optical properties. Following radiolabeling with [^68^Ga]Ga, a dual-modality radionuclide-fluorescence probe ([^68^Ga]Ga-PSMA-DF) was successfully constructed. In vitro cellular uptake studies demonstrated that 22Rv1 cells had relatively high uptake of the probe, reaching 7.34 ± 0.55 IA%/10^6^ cells at 120 min. In contrast, PC-3 cells and blocked 22Rv1 cells displayed minimal uptake, confirming the specific targeting ability of the probe. In vivo evaluations were conducted on tumor-bearing mice using micro-PET/CT and NIRF imaging. The results revealed that [^68^Ga]Ga-PSMA-DF achieved high specific tumor accumulation in 22Rv1 xenografts, with the peak tumor uptake (SUVmax = 1.748 ± 0.132) and tumor-to-muscle ratio (11.542 ± 1.511) observed at 120 min. Notably, high-contrast fluorescence imaging was also achieved at later time points, yielding a tumor-to-background ratio (TBR) of 6.559 ± 1.415 at 48 h. Notably, ex vivo biodistribution data were consistent with in vivo imaging findings. **Conclusions**: This preclinical study demonstrates that [^68^Ga]Ga-PSMA-DF exhibits high and specific uptake in PCa models, supporting its potential as a dual-modality tracer for both PET/CT imaging and real-time intraoperative fluorescence guidance during PCa surgery.

## 1. Introduction

Prostate cancer (PCa) is among the most common malignancies of the male genitourinary system, with a steadily increasing incidence in recent years [1]. Early detection and timely intervention are crucial for improving clinical outcomes. Radical prostatectomy (RP) remains a cornerstone therapy for localized and selected locally advanced PCa. With continuous advances in surgical techniques, laparoscopic and robot-assisted laparoscopic RP have been widely adopted in clinical practice [2,3]. Despite these technical improvements, accurate intraoperative identification of tumor boundaries, lymph node metastases, and small satellite lesions from normal tissues remains challenging, as malignant tissues cannot be reliably distinguished from surrounding normal structures by visual inspection alone. This often leads to incomplete tumor resection and positive surgical margins (PSMs), which are recognized as an adverse pathological feature and a significant risk factor for biochemical recurrence [4]. Consequently, the prevention of PSMs represents a major unmet clinical need in PCa surgery [5]. Therefore, there is a pressing need to develop an intraoperative navigation tool that enables real-time, precise tumor localization and clear visualization of tumor margins.

In recent years, fluorescence imaging, owing to its high sensitivity, has been increasingly utilized for the detection of malignant tumors. When seamlessly integrated into modern surgical approaches, its portability and real-time feedback have facilitated its clinical adoption for precise intraoperative guidance [6,7,8]. The strategy relies on the selective accumulation of fluorescent agents in tumor tissues to enable real-time tumor visualization. Nevertheless, the limited tissue penetration depth of near-infrared fluorescence (NIRF) imaging constrains its utility for preoperative tumor detection. Positron emission tomography (PET), by contrast, offers deeper tissue penetration and high sensitivity, effectively addressing the limitations of NIRF. However, PET involves ionizing radiation and employs short-lived radionuclides, making it unsuitable for intraoperative use. Conversely, NIRF imaging excels in delivering real-time intraoperative feedback. Thus, a dual-modal imaging strategy that combines PET and NIRF can synergistically integrate preoperative lesion assessment with intraoperative tumor visualization, offering an optimal solution to enhance surgical precision in PCa management [9].

PSMA is a type II membrane protein that is overexpressed on the surface of PCa cells and serves as a specific molecular marker for the disease [10,11,12]. Small-molecule inhibitors targeting PSMA, primarily based on glutamate-urea structures and their analogs (Glu-Urea-R), represent the most extensively studied and clinically applied PSMA ligands [13,14]. Compared with antibodies, which may trigger immune reactions and exhibit slow background clearance, PSMA-ligand-based probes demonstrate improved pharmacokinetic properties and safety, and have been continuously developed for PET assessment of PCa [15,16,17,18]. PSMA has emerged as both a diagnostic and therapeutic target in the era of targeted radionuclide therapy (TRT), particularly in peptide receptor radionuclide therapy (PRRT). The clinical efficacy of [^177^Lu]Lu-labeled, PSMA-targeted radionuclide therapy has been well established, most notably in the VISION trial, which demonstrated significant improvements in overall survival and radiographic progression-free survival among patients with metastatic castration-resistant prostate cancer (mCRPC) treated with [^177^Lu]Lu-PSMA-617 [19]. This landmark study established PSMA-directed radionuclide therapy as a standard-of-care option for patients who have progressed following androgen deprivation therapy and taxane-based chemotherapy. Despite these advances, a proportion of patients remain either ineligible for or unresponsive to radionuclide therapy. Mechanisms of resistance, including heterogeneous PSMA expression and acquired genetic alterations, continue to pose significant clinical challenges. For patients with localized disease, as well as for those who are not candidates for or who have progressed after systemic therapies, radical prostatectomy remains a critical curative approach. In this surgical setting, the rate of positive surgical margins is a key determinant of oncologic outcomes, as it is associated with an increased risk of biochemical recurrence and disease progression. To address this limitation, fluorescence-guided surgery (FGS) has emerged as a promising strategy to improve intraoperative visualization of tumor margins and the detection of occult lesions.

**Figure 1:**
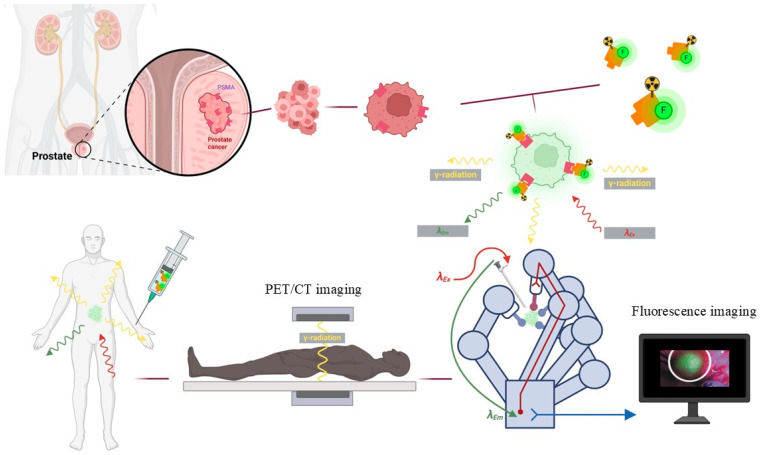
Schematic illustration of the clinical application of dual-modality PSMA-targeted probes.

Dual-modality radiolabeled-fluorescent probes derived from PSMA ligands enable both preoperative diagnosis and intraoperative localization of PCa (Figure 1). Fluorescent dyes with longer emission wavelengths offer enhanced tissue penetration and robust in vitro stability [20,21]. Among these, the novel indocyanine green derivative IR820, with fluorescence emission suitable for NIRF imaging, provides greater stability and flexibility for optical imaging applications [22]. In this study, we employed IR820 and developed a dual-modality PSMA-targeted probe, [^68^Ga]Ga-PSMA-DF, based on the Glu-Urea structure. Its tumor-targeting efficiency and in vivo pharmacokinetics were systematically evaluated through PET/NIRF imaging, demonstrating pronounced accumulation in tumors and highlighting its potential for clinical translation.

## 2. Results

### 2.1. Histological Analysis Results

**Figure 2:**
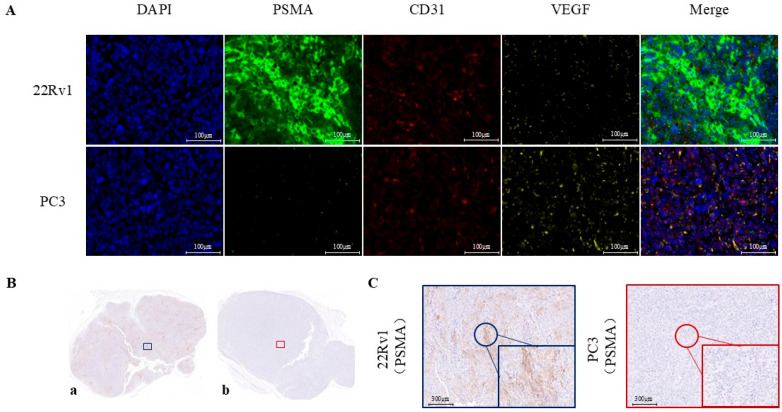
Immunohistochemistry and immunofluorescence staining images of 22Rv1 and PC-3 tumors. (**A**) Fluorescence microscopy images of PSMA (green), CD31 (red), and VEGF (yellow) in 22Rv1 and PC-3 tumor tissues; nuclei stained with DAPI (blue). (**B**) Cross-sectional views of 22Rv1 (a) and PC-3 (b) tumors. (**C**) Immunohistochemical staining of 22Rv1 (left) and PC-3 (right) tumors. Blue boxes indicate the magnified region in 22Rv1 sections, and red boxes highlight the corresponding areas in PC-3 sections.

Immunofluorescence staining and immunohistochemistry confirmed robust PSMA expression in 22Rv1 tumor tissues from 22Rv1 tumor-bearing mice, whereas PSMA levels were minimal in PC-3 tumor tissues. Both tumor types showed positive expression of vascular endothelial growth factor (VEGF) and the angiogenesis marker platelet endothelial cell adhesion molecule-1 (PECAM-1/CD31) (Figure 2).

### 2.2. Synthesis and Quality Control of the PSMA-DF Probe

**Figure 3:**
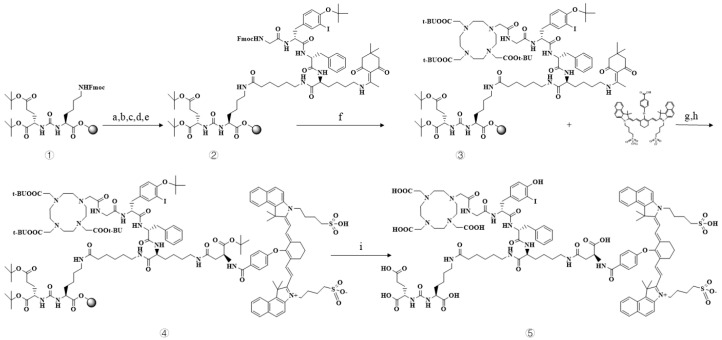
Synthetic route of PSMA-DF. Reaction conditions: a. Fmoc deprotection with 20% piperidine in DMF, followed by coupling of Fmoc-Ahx-OH using HBTU, HOBt, and DIPEA, 1 h; b. Fmoc deprotection with 20% piperidine, followed by coupling of Fmoc-Lys(Dde)-OH using HBTU, HOBt, and DIPEA, 1 h; c. Fmoc deprotection with 20% piperidine, followed by coupling of Fmoc-Phe-OH using HBTU, HOBt, and DIPEA, 1 h; d. Fmoc deprotection with 20% piperidine, followed by coupling of Fmoc-3-Iodo-Tyr(OtBu)-OH using HBTU, HOBt, and DIPEA, 1 h; e. Step ①→②: Fmoc deprotection with 20% piperidine, followed by coupling of Fmoc-Gly-OH using HBTU, HOBt, and DIPEA, 1 h; f. Step ②→③: Fmoc deprotection with 20% piperidine, followed by coupling of DOTA-(COOt-Bu)_3_ using HBTU, HOBt, and DIPEA, 1 h; g. Dde deprotection with 2% hydrazine hydrate, followed by coupling of Fmoc-Asp(OtBu)-OH using HBTU, HOBt, and DIPEA, 1 h; h. Step ③→④: Fmoc deprotection with 20% piperidine, followed by conjugation of IR820 derivative using PyBOP and DIPEA, 2 h; i. Step ④→⑤: Cleavage from resin and global deprotection using TFA, TIPS, and H_2_O, 4 h. All reactions were carried out at room temperature.

The overall synthetic route is illustrated in Figure 3.

**Figure 4:**
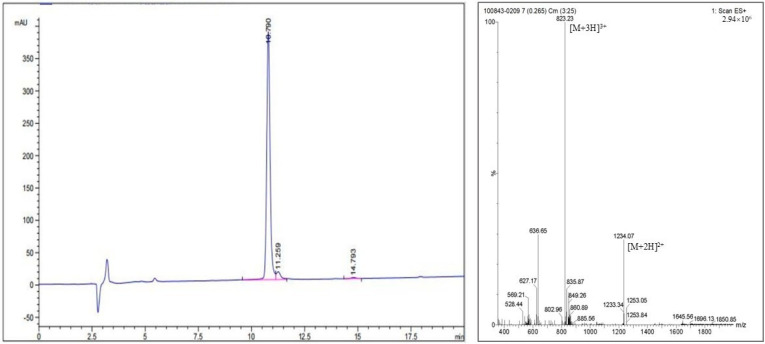
HPLC and MS analyses of PSMA-DF. The HPLC chromatogram (**left**) and the corresponding MS spectrum (**right**) are presented.

The purity and MS data for PSMA-DF are presented in Figure 4. HPLC analysis indicated that the synthesized PSMA-DF had a purity of 95.37%, with a retention time of 10.790 min, thereby meeting the required standard. MS analysis identified the primary ion peak of PSMA-DF as [M + 3H]^3+^ with an *m*/*z* value of 823.23, corresponding to an approximate molecular weight of 2466.40 (theoretical molecular weight: 2466.58). These findings confirm the successful synthesis of PSMA-DF.

### 2.3. Fluorescence Properties

**Figure 5:**
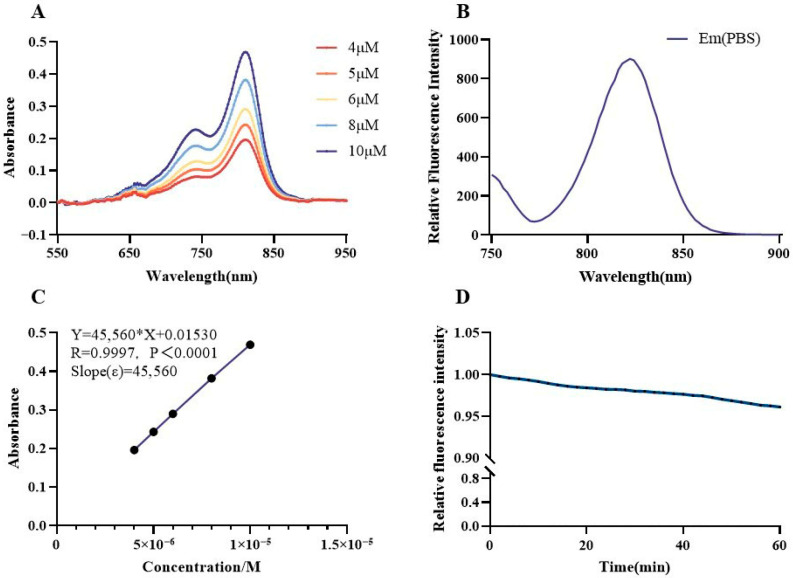
Fluorescence property measurements of PSMA-DF. (**A**) Absorbance spectra of PSMA-DF. (**B**) Emission spectra of PSMA-DF. (**C**) Linear fitting curve of PSMA-DF concentration and absorbance. The molar absorptivity (ε) corresponds to the slope of the linear equation. (**D**) Photostability profile of PSMA-DF.

PSMA-DF exhibited a primary absorption peak at approximately 810 nm, accompanied by a weaker shoulder peak at 740–746 nm with lower relative intensity. Absorbance at 810 nm increased linearly with probe concentration, demonstrating a concentration-dependent optical response (Figure 5A). The emission spectrum displayed a maximum near 820 nm (Figure 5B). The fluorescence quantum yield was measured at 1.5% (Table 1), which is consistent with typical values for near-infrared dyes in aqueous environments. Molar absorptivity (ε) was calculated as 45,560 L·mol^−1^·cm^−1^ through linear regression of absorbance versus concentration (Figure 5C), indicating strong light absorption at the excitation wavelength. Photostability was assessed under continuous irradiation at 810 nm for 60 min, during which the relative fluorescence intensity remained largely stable, with only a slight decline observed (Figure 5D).

### 2.4. In Vitro Cellular Studies

**Figure 6:**
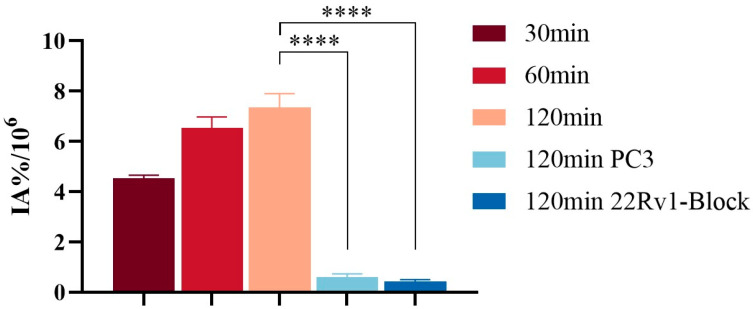
In vitro uptake of [^68^Ga]Ga-PSMA-DF in 22Rv1 and PC-3 cells. (****: *p* < 0.0001).

The cellular uptake of [^68^Ga]Ga-PSMA-DF in 22Rv1 and PC-3 cells is presented in Figure 6. In 22Rv1 cells, uptake increased progressively over time, from 4.52 ± 0.14 IA%/10^6^ cells at 30 min to 7.34 ± 0.55 IA%/10^6^ cells at 120 min. Pre-incubation with the PSMA inhibitor ZJ-43 markedly reduced uptake in 22Rv1 cells to 0.41 ± 0.09 IA%/10^6^ cells. In contrast, PC-3 cells exhibited low baseline uptake, reaching only 0.60 ± 0.13 IA%/10^6^ cells at 120 min, which was significantly lower than that observed in 22Rv1 cells (*p* < 0.05).

### 2.5. PET Imaging and Ex Vivo Biodistribution

**Figure 7:**
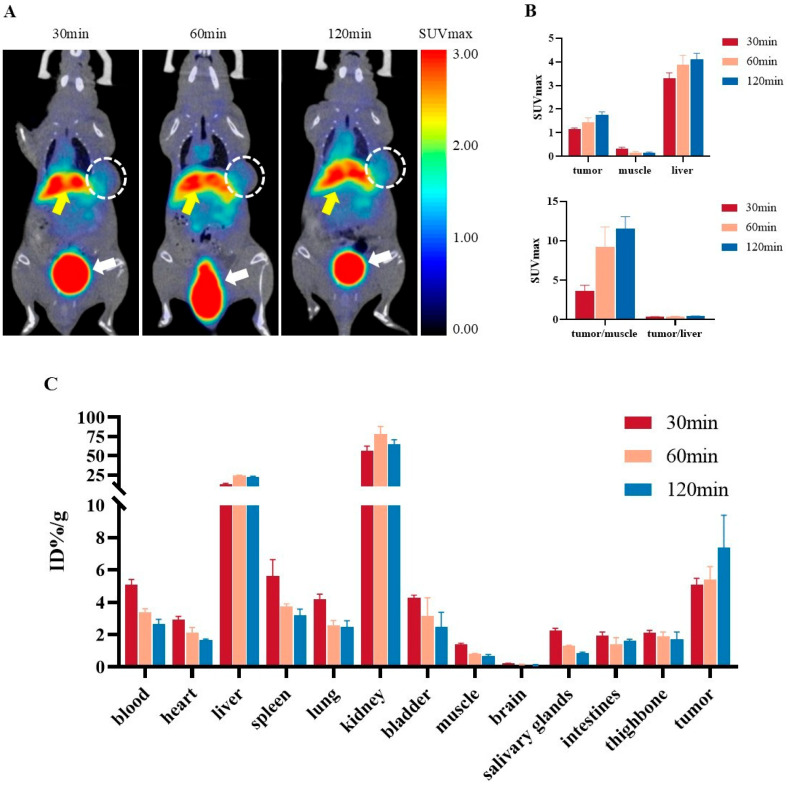
PET/CT imaging and biodistribution of [^68^Ga]Ga-PSMA-DF. (**A**) Coronal PET/CT images of 22Rv1 tumor-bearing mice, with tumors delineated by white dashed circles (yellow arrow indicates the liver; white arrow indicates the bladder). (**B**) Quantification of SUVmax values in tumors and muscle, along with tumor-to-muscle and tumor-to-liver ratios derived from in vivo PET/CT imaging. (**C**) Ex vivo tissue uptake of [^68^Ga]Ga-PSMA-DF in 22Rv1 tumor-bearing mice at 30, 60, and 120 min after intravenous injection, expressed as mean ± SD (*n* = 3).

Representative PET images of 22Rv1 tumor-bearing mice at 30, 60, and 120 min after [^68^Ga]Ga-PSMA-DF injection are shown in Figure 7A. At 30 min, clear radioactive uptake and retention were observed in the tumor. High tracer accumulation was evident in the liver, kidneys, and bladder, while other organs and tissues exhibited low background signals. Over time, background activity gradually declined, whereas radioactivity in the liver, kidneys, and bladder remained elevated. These imaging findings were consistent with the cellular uptake data observed in vitro. Semi-quantitative analysis (Figure 7B) revealed that the maximum standardized uptake value (SUVmax) in 22Rv1 tumors increased continuously from 1.138 ± 0.059 at 30 min to 1.748 ± 0.132 at 120 min. Correspondingly, the tumor-to-muscle ratio rose steadily from 3.610 ± 0.737 to 11.542 ± 1.511 over the same period.

Ex vivo biodistribution results (Figure 7C) demonstrated a progressive increase in [^68^Ga]Ga-PSMA-DF uptake in 22Rv1 tumors over 2 h, from 5.081 ± 0.390%ID/g at 30 min to 7.385 ± 2.000%ID/g at 120 min. Among all organs, the kidneys showed the highest accumulation and retained high levels throughout the observation period, followed by the liver. Uptake in other normal tissues remained low, with the brain and muscle showing the minimal values. Radioactive clearance from most tissues was observed over time. At 120 min, the tumor-to-muscle ratio reached 11.000 ± 1.435, consistent with the PET-derived quantitative data.

### 2.6. In Vivo and Ex Vivo Fluorescence Imaging

**Figure 8:**
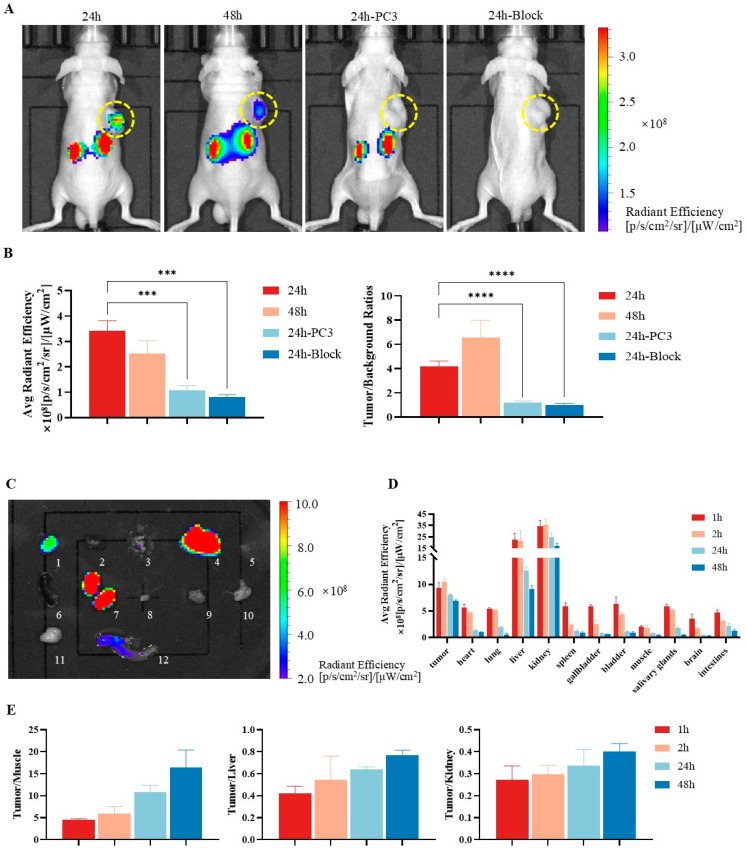
In vivo fluorescence imaging and ex vivo distribution of PSMA-DF in mice. (**A**) In vivo fluorescence images of 22Rv1 tumor-bearing mice acquired at 24 and 48 h after intravenous injection of PSMA-DF (40 µg/kg in 150 µL). Images also include PC-3 tumor-bearing nude mice (control group) and 22Rv1 tumor-bearing mice pre-treated with the PSMA inhibitor ZJ-43 (blocking group), imaged at 24 h post-injection. Tumor locations are indicated by yellow dashed circles. Fluorescence intensity was quantified as the average radiative efficiency [(photon/s/cm^2^/sr)/(µW/cm^2^)]. (**B**) Quantitative analysis of PSMA-DF uptake in tumors (left) and TBR (right). (**C**) Ex vivo distribution of PSMA-DF in 22Rv1 tumors and major organs 24 h after injection (*n* = 3): Organ labels: 1: tumor, 2: heart, 3: lung, 4: liver, 5: gallbladder, 6: spleen, 7: kidney, 8: bladder, 9: muscle, 10: salivary gland, 11: brain, 12: small intestine. (**D**) ROI-based quantification of fluorescence signals in major tissues at 1, 2, 24, and 48 h after PSMA-DF administration. Error bars represent standard deviation (*n* = 3). (**E**) Fluorescence signal intensity ratios of tumor-to-muscle (left), tumor-to-liver (middle), and tumor-to-kidney (right) at 1 h, 2 h, 24 h, and 48 h after PSMA-DF injection. Error bars represent standard deviation (***: *p* < 0.001; ****: *p* < 0.0001).

Fluorescence imaging was performed in 22Rv1 tumor-bearing mice at 24 and 48 h after intravenous administration of PSMA-DF (40 µg/kg in 150 µL) (Figure 8). At 24 h post-injection, the 22Rv1 tumors exhibited a strong fluorescence signal (3.424 ± 0.389 × 10^8^ [(photon/s/cm^2^/sr)/(µW/cm^2^)]) with a tumor-to-background ratio (TBR) of 4.197 ± 0.424. By 48 h, although the absolute fluorescence signal decreased, the TBR further increased to 6.559 ± 1.415, indicating improved contrast over time. These findings were consistent with the in vitro cellular uptake data. In PC-3 tumor-bearing mice, PSMA-DF accumulation in tumors was significantly lower than in 22Rv1 tumors (*p* < 0.05). At 24 h post-injection, no discernible tumor fluorescence was observed in PC-3 xenograft models, whereas intense signals were detected in the kidneys. The PSMA-targeting specificity of PSMA-DF was confirmed through a blocking experiment. In the 22Rv1 blocking group, animals were pre-administered with the PSMA-specific inhibitor ZJ-43 prior to PSMA-DF injection, which markedly reduced probe uptake in both tumor and kidney tissues, resulting in a TBR of only 0.988 ± 0.131 at 24 h. Additionally, ex vivo fluorescence imaging was conducted to characterize the tissue distribution of PSMA-DF. Ex vivo imaging results and quantitative data from twelve 22Rv1 tumor-bearing nude mice (*n* = 3 per group) are shown in Figure 8C–E. One hour after injection, high accumulation of PSMA-DF was observed in 22Rv1 tumors, accompanied by variable uptake in other organs. Over time, systemic clearance occurred in most normal tissues, whereas tumor retention remained pronounced, demonstrating favorable target-to-background contrast.

### 2.7. Acute Toxicity Evaluation

**Figure 9:**
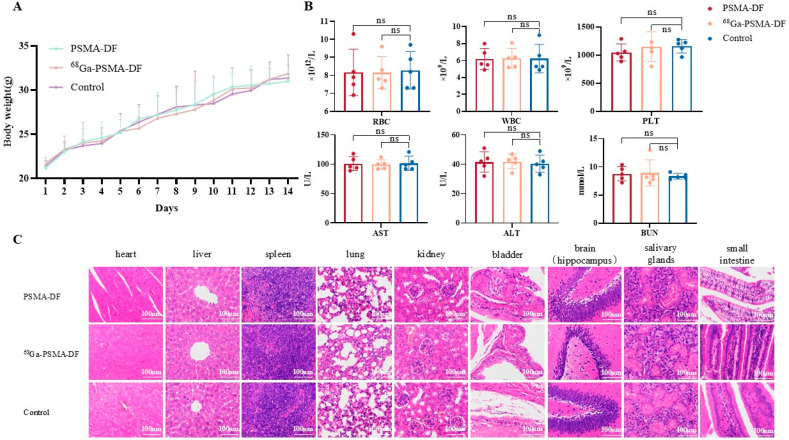
Acute toxicity evaluation of PSMA-DF. (**A**) Body weight changes in mice over 14 days following intravenous administration of [^68^Ga]Ga-PSMA-DF, PSMA-DF, or normal saline. (**B**) Hematological and biochemical parameters measured 14 days post-injection, including RBC, WBC, PLT, BUN, AST, and ALT. (**C**) H&E staining of major organs including heart, liver, spleen, lung, and kidney. (ns: not statistically significant, *p* > 0.05).

No mortality was observed in mice administered with high-dose [^68^Ga]Ga-PSMA-DF, unlabeled PSMA-DF, or normal saline over the 14-day post-injection period. Throughout the observation, no significant differences were noted among the groups in terms of dietary intake, physical activity, mental status, skin condition, or body weight changes (Figure 9A). On day 14, hematological and biochemical parameters, including RBC, WBC, PLT, BUN, AST, and ALT, showed no statistically significant differences across the groups (Figure 9B). Histopathological examination of major organs, including the heart, liver, spleen, lung, and kidney, via H&E staining revealed no apparent pathological abnormalities (Figure 9C). These findings indicate that PSMA-DF is well tolerated and exhibits a favorable safety profile, supporting its potential for further translational studies.

## 3. Discussion

In recent years, multimodal imaging has undergone rapid development and demonstrated substantial clinical value. By combining complementary information from different imaging modalities, these approaches enhance both diagnostic accuracy and therapeutic guidance. For example, PET/CT or PET/MR integrates the metabolic sensitivity of PET with the anatomical detail provided by CT or MR within a single scan. Despite these advances, single-modality tracers remain predominant at the molecular probe level. Repeated injections of different imaging agents can complicate precise image registration due to physiological variations over time. Dual-modal probes overcome this limitation by allowing synchronized detection with a single administration, thereby offering improved clinical applicability compared with unimodal agents.

In surgical practice, optical imaging has gained increasing attention for intraoperative guidance due to its real-time visualization capability and operational flexibility [6,7,8]. In RP, fluorescence imaging has been actively explored in both preclinical and clinical settings to assist in delineating tumor margins and detecting metastatic lymph nodes. Indocyanine green (ICG) has been widely employed for vascular and lymphatic mapping owing to its rapid systemic clearance and favorable biocompatibility, demonstrating utility in intraoperative navigation and postoperative assessment [23,24]. Manny et al. [25] administered ICG into the prostatic lobes of 50 PCa patients during surgery and observed fluorescence signals in the vas deferens, obturator nerve, and neurovascular bundles within 10 min. Notably, all patients with pathologically confirmed positive lymph nodes consistently exhibited strong near-infrared fluorescence during surgery. Nevertheless, as a non-targeted agent, ICG lacks molecular specificity, and its passive accumulation often results in high background signals, limiting both image contrast and detection reliability. To address this, Kularatne et al. [26] developed a PSMA-targeted near-infrared fluorescent probe. In PCa models, FGS with this probe enabled real-time identification of metastatic lymph nodes and tumor boundaries, facilitating more complete resection and potentially improving oncologic outcomes. Several recent studies have investigated dual-modal PSMA-targeting probes that combine PET and fluorescence imaging capabilities. Lozada et al. [27] reported the synthesis and preclinical evaluation of two dual-mode tracers incorporating an organotrifluoroborate moiety for one-step [^18^F]F-radiofluorination, together with fluorescein moieties (FAM and FITC) and pharmacophores derived from PSMA-617 and PSMA-1007. This approach achieved high molar activity (42–167 GBq/μmol) and demonstrated tumor uptake of 7–17% ID/g in LNCaP and 22Rv1 xenografts, along with strong ex vivo fluorescence visualization. Similarly, studies by Baranski et al. [28] and Chen et al. [29] described dual-labeled inhibitors derived from PSMA ligands for preoperative PET imaging and fluorescence-guided surgery.

We developed a PSMA-targeted fluorescent probe, PSMA-DF, based on the PSMA ligand scaffold. To enable dual-modality functionality, a metal chelator (DOTA) was incorporated into the PSMA-DF structure. The core design principle was that a single molecular entity can simultaneously carry both a radionuclide and a near-infrared fluorophore, thus conferring the unique capability of “one injection, dual tracing.” This strategy aims to seamlessly bridge preoperative diagnosis with intraoperative navigation. Compared to conventional approaches that rely on multi-probe combinations or hardware-based image fusion, this single-entity dual-modal probe ensures consistent biodistribution of diagnostic and navigational signals, thereby eliminating inter-agent discrepancies, minimizing registration errors, simplifying clinical workflows, and providing a solid molecular platform for theranostic integration.

In this study, we developed a dual-modal radionuclide-fluorescence probe, [^68^Ga]Ga-PSMA-DF, by conjugating the nonspecific fluorescent dye IR820 with a PSMA-targeting ligand, thereby integrating PET and NIRF imaging capabilities. The deep tissue penetration of gamma radiation enables early tumor detection and precise preoperative localization, while the high spatial resolution of NIRF imaging supports real-time intraoperative visualization of tumor boundaries. The tumor-targeting efficacy of [^68^Ga]Ga-PSMA-DF was evaluated through comprehensive in vitro and in vivo validations. In vitro cellular uptake studies revealed a time-dependent increase in probe accumulation in 22Rv1 cells over 120 min, whereas uptake remained minimal in PC-3 cells and in ZJ-43-blocked 22Rv1 cells, confirming PSMA-specific binding. In vivo evaluations included micro-PET and NIRF imaging. Micro-PET imaging showed progressive accumulation of the probe in 22Rv1 tumors over 120 min, with rapid clearance from muscle tissue, leading to a continuously increasing tumor-to-muscle ratio. NIRF imaging demonstrated gradual enhancement of tumor-to-background contrast over 48 h, with background signals nearly undetectable at 48 h post-injection, while strong fluorescence persisted in 22Rv1 tumors, defining an optimal window for surgical intervention. In contrast, negligible fluorescence signals were detected in PC-3 tumors and in the blocking group. Ex vivo biodistribution data corroborated the imaging findings. It is important to note that the observed hepatobiliary uptake of the probe may limit the detection of liver metastases using PET imaging, as high physiological liver background could obscure metastatic lesions. However, the primary clinical application of this study is fluorescence-guided surgery (FGS) for primary prostate cancer, aiming to reduce positive surgical margins during radical prostatectomy or pelvic lymph node dissection. In this context, the surgical field is confined to the pelvis, and hepatic uptake does not interfere with intraoperative visualization. Furthermore, patients with established liver metastases typically present with advanced systemic disease and are generally not candidates for curative-intent radical prostatectomy, the procedure in which FGS would be applied. Therefore, while the hepatic clearance profile may limit PET-based detection of liver metastases, it does not compromise the translational potential of the fluorescent probe for primary prostate cancer surgery. A critical consideration for clinical translation is the expected specific activity of the radiotracer in humans, which directly influences the total probe mass administered. In preclinical studies, the injected dose often contains a higher molar amount of the probe due to carrier-added [^68^Ga]Ga or relatively low specific activity. In clinical settings, specific activity is substantially higher, resulting in a significantly lower total probe mass. At these lower concentrations, the question arises whether fluorescence signals remain detectable with intraoperative imaging systems. Several factors support the feasibility of fluorescence detection under clinical dosing conditions. First, the PSMA-targeting ligand exhibits high binding affinity (low nanomolar Ki), ensuring efficient accumulation in PSMA-expressing tissues even at low administered mass. Second, the fluorophore employed has a high molar extinction coefficient and quantum yield, enabling sensitive detection with modern fluorescence imaging systems capable of visualizing nanomolar to picomolar concentrations in tissue. Third, the delayed imaging window (24–48 h post-injection) allows substantial clearance of non-specifically bound probe, maximizing the TBR, a critical determinant of signal detection even when absolute fluorescence intensity is modest. From a workflow perspective, the dual-modal design provides a distinct advantage in a “see-and-treat” paradigm. Patients first undergo PET/CT imaging with the [^68^Ga]Ga-labeled probe for preoperative lesion localization and surgical planning. The same probe, present at the time of surgery (24–48 h later), then serves as a fluorescence guide without requiring a second injection. This approach streamlines clinical workflow, reduces patient radiation exposure from additional injections, and ensures perfect spatial correlation between preoperative PET signals and intraoperative fluorescence. Even if the fluorescence signal is somewhat attenuated at clinical doses, the ability to use a single molecular probe for both diagnosis and surgery without re-dosing represents a significant logistical and clinical advantage. Acute toxicity of the probe was assessed following high-dose intravenous administration. No signs of acute toxicity were observed over a 14-day observation period. Monitoring of body weight, hematological and biochemical parameters, and histopathological examination of major organs revealed no abnormalities. These results indicate that [^68^Ga]Ga-PSMA-DF possesses a favorable safety profile, supporting its potential for clinical translation.

## 4. Materials and Methods

### 4.1. General

All chemicals were obtained from commercial suppliers and used without further purification. Organic solvents and reagents, including dichloromethane (DCM), N,N-dimethylformamide (DMF), O-(benzotriazol-1-yl)-N,N,N′,N′-tetramethyluronium hexafluorophosphate (HBTU), 1-hydroxybenzotriazole (HOBt), and N,N-diisopropylethylamine (DIPEA), were purchased from Chinapeptides Co., Ltd. (Suzhou, China). Fluorescent dyes were acquired from Macklin Biochemical Technology Co., Ltd. (Shanghai, China).

### 4.2. Cell Lines and Culture Conditions

Human PCa cell lines 22Rv1 and PC-3 were obtained from GuYan Biotech Co., Ltd. (Shanghai, China). Both cell lines were maintained in RPMI-1640 medium (Gibco, Life Technologies, Grand Island, NY, USA) supplemented with 10% fetal bovine serum (FBS) and 1% penicillin-streptomycin (P/S), and incubated at 37 °C in a humidified atmosphere containing 5% CO_2_.

### 4.3. Animal Preparation

All animal experiments were conducted in accordance with the guidelines approved by the Ethics Committee of the General Hospital of the Chinese People’s Liberation Army and relevant institutional review boards. Experimental animals were obtained from Charles River Laboratories (Beijing, China). Male BALB/c nude mice (3–4 weeks old, 16–18 g) and male ICR mice of the same age range (20–25 g) were used in this study. Animals were housed under specific pathogen-free (SPF) conditions with a standard 12-h light/dark cycle, controlled temperature, and ad libitum access to food and water. A 7-day acclimatization period was allowed prior to experimental procedures.

To establish 22Rv1 (or PC-3) tumor xenografts, a suspension of 22Rv1(or PC-3) cells (approximately 5 × 10^6^ cells in 100 µL) was subcutaneously injected into the right shoulder of BALB/c nude mice. Tumor growth was monitored using calipers, and tumor volume was calculated as V = 1/2 × long diameter × (short diameter)^2^. Imaging studies were initiated when tumor volumes reached 200–300 mm^3^. PC-3 cell xenografts were generated following the same procedure.

### 4.4. Histological Analysis

Immunohistochemistry and immunofluorescence staining were performed as detailed in the Figure 4 to evaluate PSMA expression in xenograft tumors derived from 22Rv1 and PC-3 models. Histological findings were used to validate the accuracy of tumor localization observed in PET and fluorescence imaging.

### 4.5. Synthesis and Quality Control of the PSMA-DF Probe

The PSMA-DF probe was synthesized using solid-phase peptide synthesis. Briefly, a predetermined amount of Resin-EUK was loaded into a solid-phase synthesis tube and allowed to rehydrate through sequential washing with DCM (3 × 2 mL, 5 min per wash), followed by three washes with DMF (3 × 2 mL, 2 min per wash). The Fmoc protecting group at the amino terminus was removed using 20% piperidine in DMF (2 × 2 mL, 10 min each), after which the resin was washed with DMF (3–5 × 2 mL, 2 min each). For amino acid coupling, side-chain-protected amino acids (three equivalents relative to resin loading) were activated in DMF with HBTU (3.6 equivalents), HOBt (3.6 equivalents), and DIPEA (7.2 equivalents) and added to the resin. The reaction mixture was stirred at room temperature for 1 h, after which the resin was rinsed again with DMF (3–5 × 2 mL, 2 min each). Upon completion of the peptide assembly, the crude product was cleaved from the resin using a cleavage cocktail of trifluoroacetic acid (TFA, 4.5 mL), triisopropylsilane (TIPS, 250 µL), and water (250 µL), followed by incubation at room temperature overnight. The resulting solution was filtered, and the resin was rinsed with an additional 2 mL of TFA. The combined filtrates were concentrated under reduced pressure to remove most of the TFA, and the residue was subsequently purified by preparative high-performance liquid chromatography (HPLC) and lyophilized to yield the final probe. The structure of the product was confirmed by HPLC and mass spectrometry (MS) analysis.

Product purity was assessed by HPLC with UV detection at 220 nm, using a Kromasil 100-5 C18 reversed-phase column (4.6 × 250 mm, 5 µm). The mobile phase initially comprised 40% solvent A (1% TFA in acetonitrile) and 60% solvent B (1% TFA in water), and was gradually changed to 100% solvent A and 0% solvent B over 20 min at a flow rate of 1.0 mL/min. The identity of PSMA-DF was confirmed by MS.

### 4.6. Preparation of Radiotracers

The PSMA-DF probe was synthesized as described above. [^68^Ga]Ga was eluted from a germanium-68/gallium-68 generator (Department of Nuclear Medicine, Chinese People’s Liberation Army General Hospital, Beijing, China). [^68^Ga]Ga was obtained by eluting a germanium-68/gallium-68 generator with 2 mL of 0.05 M HCl. The eluate was added to a reaction vial containing PSMA-DF (50 µg) dissolved in 1 M sodium acetate buffer (130 μL). The mixture was heated at 90 °C for 10 min with manual shaking. After cooling to room temperature, the reaction mixture was diluted with 5 mL of water and passed through a pre-conditioned C18 cartridge (Waters, pre-conditioned with 5 mL ethanol followed by 10 mL water). The cartridge was washed with 10 mL of water, and the product was eluted with 0.5 mL ethanol. Radiochemical purity was determined by radio-TLC (or radio-HPLC) and was consistently >95%. The decay-corrected radiochemical yield (RCY) was 70%. Radiolabeling of PSMA-DF with [^68^Ga]Ga was then performed to obtain the final product, designated as [^68^Ga]Ga-PSMA-DF.

#### Fluorescence Properties

PSMA-DF absorption spectra were recorded using a fluorescence spectrophotometer (Shimadzu RF-6000, Shimadzu, Kyoto, Japan). Fluorescence emission spectra and fluorescence quantum yields were measured with a time-resolved fluorescence spectrometer (FLS 980, Edinburgh Instruments, Livingston, UK). The probe was diluted with phosphate-buffered saline (PBS) to final concentrations of 4, 5, 6, 8, and 10 µM, and UV–vis absorption spectra were acquired over the wavelength range of 550–950 nm. The emission spectrum of PSMA-DF at 10 µM was recorded between 750 and 900 nm. The absolute fluorescence quantum yield (Φ, %) at 5 µM was determined using a Hamamatsu Quantaurus-QY C11347-11 absolute quantum yield spectrometer (Hamamatsu, Shizuoka, Japan). Molar absorptivity (ε, L·mol^−1^·cm^−1^) was calculated from five PBS dilutions (3 mL each) based on the Beer–Lambert law. Photostability was assessed by continuous irradiation at 810 nm for 60 min under ambient conditions.

### 4.7. In Vitro Cellular Studies

In vitro cellular uptake and blocking assays were performed using 22Rv1 and PC-3 cells. Cells were seeded in 24-well plates (5 × 10^4^/well) and incubated in a humidified incubator at 37 °C with 5% CO_2_ for 24 h prior to experiments. Two hours before the assay, the medium was replaced to ensure optimal conditions. For uptake studies, 22Rv1 cells were incubated with [^68^Ga]Ga-PSMA-DF (3.7 × 10^4^ Bq in 100 µL, diluted in 900 µL RPMI-1640) for 30, 60, and 120 min, while PC-3 cells were incubated under the same conditions for 120 min only. After incubation, cells were washed twice with ice-cold PBS (1000 µL) to remove unbound probe, followed by lysis with 500 µL of 0.5 mol/L NaOH. The resulting lysates were collected for subsequent analysis. Radioactivity was quantified using a γ-counter (Gamma counter, Hidex, Turku, Finland), and cellular uptake was calculated as internalized activity per 10^6^ cells (IA%/10^6^). In blocking experiments, 22Rv1 cells were pre-incubated with the PSMA-specific inhibitor ZJ-43 [30,31,32] for 20 min prior to the addition of [^68^Ga]Ga-PSMA-DF to assess binding specificity.

### 4.8. PET Imaging and Ex Vivo Biodistribution

22Rv1 tumor-bearing mice were anesthetized with 2% sevoflurane and positioned in a PET scanner (SUPER-NOVA, Pingsheng Technology, Wuxi, China). Whole-body PET/CT imaging was performed in 22Rv1 tumor-bearing mice. Five mice were randomly selected and intravenously administered [^68^Ga]Ga-PSMA-DF (3.7 × 10^6^ Bq in 150 µL) via the tail vein. PET/CT scans were acquired at 30, 60, and 120 min post-injection. During image acquisition, animals were maintained under continuous inhalational anesthesia with 1.0% isoflurane in air.

To characterize the in vivo biodistribution of [^68^Ga]Ga-PSMA-DF, an ex vivo biodistribution study was conducted in 22Rv1 tumor-bearing mice. Nine mice were randomly assigned to three groups (*n* = 3 per group) and euthanized at 30, 60, or 120 min after intravenous administration of [^68^Ga]Ga-PSMA-DF (9.25 × 10^5^ Bq in 150 µL). Tumors and major tissues, including blood, heart, liver, spleen, lung, kidney, bladder, muscle, brain, salivary gland, small intestine, and bone, were carefully harvested, weighed, and analyzed for radioactivity using a γ-counter. Tissue uptake was calculated as percentage of the injected dose per gram of tissue (%ID/g) after decay correction.

### 4.9. In Vivo and Ex Vivo Fluorescence Imaging

In vivo fluorescence imaging was performed in tumor-bearing nude mice using PSMA-DF. Mice were placed into a small-animal live fluorescence imaging system (IVIS Lumina III, Perkin-Elmer, Shelton, CT, USA). Fluorescence signals were collected at specific excitation and emission wavelengths. All images were acquired using identical acquisition parameters, including exposure height (1.5 cm), fluor lamp level (high), and exposure time (1 s), and were processed uniformly. Fluorescence intensity was quantified as the average radiative efficiency [(photon/s/cm^2^/sr)/(µW/cm^2^)]. For time-course imaging, 22Rv1 tumor-bearing mice (*n* = 5) were injected intravenously with PSMA-DF (40 µg/kg in 150 µL), and in vivo imaging was conducted at 24 and 48 h post-injection. PC-3 tumor-bearing mice (*n* = 5) were imaged at 24 h post-injection. In the blocking group, 22Rv1 tumor-bearing mice (*n* = 5) were pre-treated with the PSMA-specific inhibitor ZJ-43 (50 mg/kg, i.v.) 30 min prior to PSMA-DF injection, followed by imaging at 24 h.

For ex vivo fluorescence imaging, twelve additional 22Rv1 tumor-bearing mice were randomly assigned to four groups (*n* = 3) and euthanized at 1, 2, 24, and 48 h after intravenous injection of PSMA-DF. Major organs and tumors were harvested and subjected to fluorescence imaging using an in vivo imaging system. Regions of interest (ROIs) were delineated, and average fluorescence intensities were quantified for subsequent analysis.

### 4.10. Acute Toxicity Evaluation

Healthy male ICR mice were randomly assigned to three groups (*n* = 5): [^68^Ga]Ga-PSMA-DF (3.7 × 10^7^ Bq in 150 µL), PSMA-DF (50 mg/kg in 150 µL), and normal saline (150 µL, control group), all administered via tail vein injection. Mice were observed for 14 days, during which body weight was recorded at regular intervals, before being sacrificed at the end of the study. Blood samples were collected for hematological and biochemical analysis, including red blood cell count (RBC), white blood cell count (WBC), platelet count (PLT), blood urea nitrogen (BUN), aspartate aminotransferase (AST), and alanine aminotransferase (ALT). Major organs were subsequently harvested and processed for histopathological examination using hematoxylin and eosin (H&E) staining.

### 4.11. Data Processing and Statistical Analysis

Quantitative data are presented as mean ± standard deviation (SD). Group comparisons were assessed using Student’s *t*-test or one-way analysis of variance (ANOVA), as appropriate. A *p* value < 0.05 was considered statistically significant. Statistical analyses were conducted using SPSS (version 29.0) and GraphPad Prism (version 9).

## 5. Conclusions

The novel dual-modal probe [^68^Ga]Ga-PSMA-DF exhibits high sensitivity, specific tumor targeting, and a favorable safety profile. In vivo imaging demonstrates pronounced tumor uptake and excellent tumor-to-background contrast. These findings suggest that [^68^Ga]Ga-PSMA-DF holds promise for integrating preoperative PET-based diagnosis with intraoperative NIRF-guided surgery. Future work will focus on evaluating the feasibility of its clinical translation.

## Figures and Tables

**Table 1:** Absorbance (λAbs), emission (λEm) maxima, and quantum yield of PSMA-DF.

Probe	PSMA-DF
λAbs max (nm)	810
λEm max (nm)	820
Φ (%)	1.5

## Data Availability

The original contributions presented in this study are included in the article. Further inquiries can be directed to the corresponding authors.
